# Adherence to Asthma Controller Therapy Among Children in Majmaah City, Saudi Arabia

**DOI:** 10.7759/cureus.14633

**Published:** 2021-04-22

**Authors:** Abdullah M AlOlayan, Meshary A Alhammad, Abdulrahman A Almutairi, Mazin T Alshammari, Sultan Albuhairi

**Affiliations:** 1 ‏Department of Pediatrics, College of Medicine, Majmaah University, Majmaah, SAU; 2 Medicine, College of Medicine, Majmaah University, Majmaah, SAU; 3 Department of Pediatrics - Allergy and Immunology Section, King Faisal Specialist Hospital and Research Center, Riyadh, SAU

**Keywords:** key words : adherence, asthma, majmaah

## Abstract

Background and objective

Improving adherence to asthma medications may prevent asthma exacerbation, which is associated with a decline in lung function. The purpose of this study was to assess the adherence to asthma controller therapy and the factors that might influence the level of adherence among asthmatic children.

Materials and methods

We conducted a prospective observational study at the King Khalid Hospital in Majmaah, Saudi Arabia between January and April 2020; the study was conducted among children aged 1-14 years with a diagnosis of asthma. The data collected when available included age and gender by using a pre-tested questionnaire that contained four validated items, and the respondents were the parents of the affected children. Due to the coronavirus disease 2019 (COVID-19) pandemic, the data collection was performed via phone calls to ensure safety. Informed consent was obtained from the parents.

Results

We analyzed 152 asthmatic children to evaluate their adherence to asthma controller therapy. The majority of the children were males (60%). Asthma was most prevalent in the age group of 6-10 years (40.1%), followed by the age group of two to five years (32.9%). We found that the majority of the patients had poor adherence to asthma medication (83.6%) while the remaining had good adherence (16.4%). The total mean score for adherence to medication therapy was 5.16 (SD: 2.3).

Conclusions

The present study showed that the overall adherence level to the asthma controller therapy was poor among children. Larger, nationwide studies are needed to assess the adherence to asthma medications and implement interventions that can improve the same.

## Introduction

Asthma is becoming one of the most devastating chronic diseases affecting populations globally. It affects the respiratory tract and damages it in several ways, ranging from airflow obstruction, mucus stacking, and blocking the bronchioles to causing an increase in the response of the bronchioles [1]. Asthma is a widespread condition with heterogeneous etiologies including genetic and environmental factors such as air pollutants [2-4]. The World Health Organization (WHO) in 2016 estimated that around 339 million individuals are suffering from asthma worldwide, and the condition is more prevalent among children. The effects of asthma are not limited to patients, but also felt by their families, and its impact extends to the community in general if we take into account missed working days and absence from school, poor quality of life, as well as the frequent visits to the emergency department and hospital stays [5-7]. According to the International Study of Asthma and Allergies in Childhood (ISAAC) Phase Three survey, which was conducted among 80,0000 children in 97 different countries, the presence of asthma symptoms ranged from 0.8% to 32.6% among those aged 13-14 years, and asthma symptoms are likely to be more prevalent in wealthy countries but more severe in less wealthy ones [3]. In Saudi Arabia, the condition affects about two million patients including children and adults, and many studies that were conducted in Saudi Arabia have reported that asthma prevalence is on the rise in the country [7]. The healthcare system in Saudi Arabia is primarily a public-based system, and the Ministry of Health provides about 60% of the healthcare services, while the remaining is provided by the private sector [8].

Asthma exacerbations are more common among children and may be associated with a decline in lung function, especially in cases of recurrent severe exacerbations early in life [9]. Hence, it is important to implement strategies that may prevent asthma exacerbation, such as ensuring adherence to asthma medications. The adherence to asthma treatment has been generally poor and a major concern despite the fact that numerous studies have focused on devising methods to improve adherence [10]. It is estimated that the mean proportion of adherence to asthma medications among children ranges from 19 to 64%, in terms of the adherence to prescribed doses of inhaled corticosteroids [11]. Nonadherence to asthma medication is thought to be linked to a number of modifiable risk factors such as low parental expectations regarding symptom control, inappropriate parental attitude towards asthma control guidelines, non-existence of a family routine to administer the medications, and the overall concern about asthma medications. Therefore, adherence to asthma controller therapy is likely to be improved with the implementation of interventions that target those risk factors, which would have a favorable impact on asthma control in general [12]. On the other hand, maternal asthma, prematurity, and race of the children are non-modifiable risk factors that might lead to nonadherence and asthma exacerbation [13-15]. Multiple studies have indicated that adherence level may influence the patient’s health either positively or negatively and poor asthma control may result in a poorer quality of life among children [16], and enhancing the communication between the physician and the patient may improve the patient’s adherence level, which would lead to good outcomes regarding the general condition [17]. In light of these factors, we sought to assess the adherence to asthma controller therapy prescribed by the physicians among children with asthma in a secondary care center in Saudi Arabia. This the first study to be conducted in this region to examine the level of adherence to asthma therapy.

## Materials and methods

We conducted a prospective observational study at the King Khalid Hospital in Majmaah, Saudi Arabia (a secondary care referral center in the Riyadh Province with a 250-bed capacity) between January and April 2020. We included patients aged 1-14 years, who were seen in the outpatient department with a diagnosis of asthma, based on the usage of asthma inhalers.

The data was collected by calling up parents after outpatient visits; a minority of the parents did not answer the calls while only one parent refused enrollment in the study. The data collected included age group, gender, and causes of poor adherence to asthma inhalers, and we used a pre-tested questionnaire [18] that contained four validated items, and the respondents were the parents of the affected children. Due to the coronavirus disease 2019 (COVID-19) pandemic, the data collection was performed via phone calls to ensure safety. Informed consent was obtained from the parents.

This study was approved by the Central IRB, Ministry of Health, Kingdom of Saudi Arabia (approval number: 20-53 E).

Scoring

The assessment of adherence to asthma controller therapy was performed by using the Pediatric Inhaler Adherence Questionnaire (PIAQ), a validated four-item questionnaire that measures the adherence of inhaled asthma therapy in children with asthma (Table 2). The total score for adherence to asthma controller therapy was obtained by analyzing the answers to all questions. The total score was then divided into two groups, categorized as good adherence when the score was 3 or above, while a score of less than 3 indicated poor adherence.

Statistical analysis

Data were presented using numbers, percentages, and mean and standard deviation, as appropriate. The relationship between asthma medication adherence and the basic demographic data of the patients was analyzed using the chi-squared test. A p-value of <0.05 was considered statistically significant. All data analyses were performed using SPSS Statistics version 21 (IBM, Armonk, NY).

## Results

Data of a total of 152 children with asthma were analyzed during the study period to evaluate their adherence to asthma controller therapy. Nearly 60% were males. Asthma was most prevalent in the age group of 6-10 years (40.1%), followed by the age group of two to five years (32.9%) (Table 1).

**Table 1:** Basic demographic data of the patients with asthma (n=152)

Study data	N (%)
Gender	
Male	91 (59.9%)
Female	61 (40.1%)
Age group	
<2 years	31 (20.4%)
2–5 years	50 (32.9%)
6–10 years	61 (40.1%)
11–14 years	10 (6.6%)

The adherence to asthma controller therapy was measured during the last 15 days from the patients' last clinic visit. We found that nearly two-thirds (64.5%) of the parents reported that their children had missed either puffs of fluticasone propionate or fluticasone-salmeterol inhalers. The number of missed puffs varied among those children; 40 out of 98 (41%) had missed >15 puffs, whereas 37% had missed one to two puffs and the remaining participants were in between. Additionally, 97.4% of the children did not consume any extra inhalers. Furthermore, the majority of the parents (88.8%) reported that they had not given any dose of inhalers to any person other than their children. Moreover, 20.4% of the parents reported that they had dispensed more than 15 puffs in the air to check whether the inhaler was empty or not; 17.8% had dispensed one to five puffs, 5.9% had dispensed 6-10 puffs, while half of the parents did not dispense any puff. The total mean score for adherence to medication therapy was 5.16 (SD: 2.30) (Table 2).

**Table 2:** Assessment of adherence to asthma controller therapy during the past 15 days (n=152)

Study data	N (%)
Number of puffs that your child did not take of the fluticasone propionate or fluticasone-salmeterol inhalers	
None	54 (35.5%)
1–5	36 (23.7%)
6–10	16 (10.5%)
11–15	06 (3.9%)
>15	40 (26.3%)
Number of extra inhalers that your child took	
None	148 (97.4%)
1–5	3 (2.0%)
6–10	0
11–15	0
>15	1 (0.70%)
Number of doses of the inhaler given to any person other than your child	
None	135 (88.8%)
1–5	8 (5.3%)
6–10	1 (0.70%)
11–15	8 (5.3%)
>15	0
Number of puffs of the inhaler dispensed in the air to check if it was empty or working correctly, or for any other reason	
None	80 (52.6%)
1–5	27 (17.8%)
6–10	9 (5.9%)
11–15	5 (3.3%)
>15	31 (20.4%)
Total score, mean ± SD	5.16 ± 2.30

**Figure 1:**
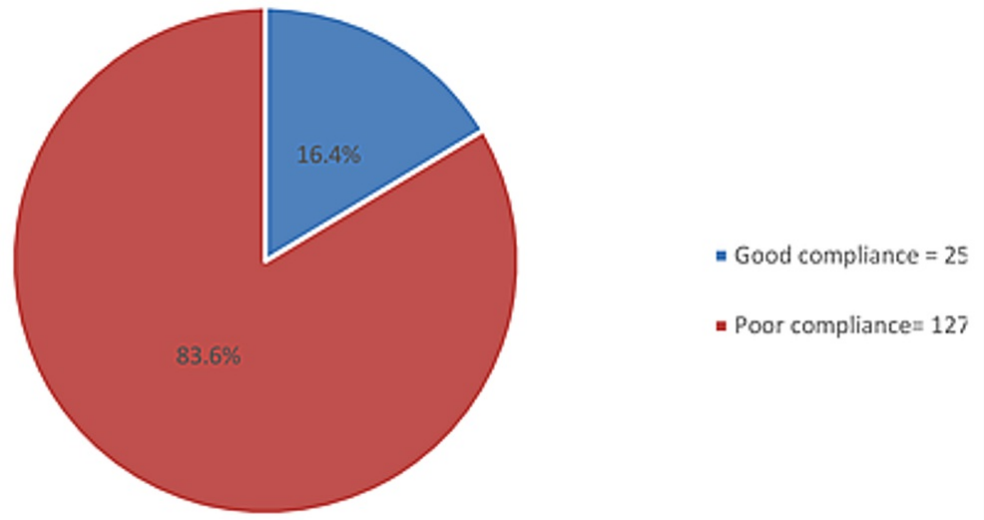
Level of adherence to asthma medication

We found that majority of the patients had poor adherence to asthma medication (83.6%) while the remaining showed good adherence (16.4%) (Figure 1). The reasons for reduced adherence are presented in Table 3; the following statement received the most number of responses, “Because you forgot to give it to your child” (55.1%), followed by “Because you felt your child was better” (22.4%), and “Because you thought your child did not need it anymore” (18.4%).

**Table 3:** Reasons for reduced adherence to metered-dose inhaler use as reported by parents of children with asthma

Reasons	N (%)
Because you forgot to give it to your child	54 (55.1%)
Because you felt your child was better	22 (22.4%)
Because the inhaler is so expensive	0
Because you thought your child did not need it anymore	18 (18.4%)
Because you were afraid that your child would become dependent on the inhaler	1 (1.0%)
Because you were afraid the inhaler would be harmful to your child	7 (7.1%)
Because of the side effects that the inhaler would cause to your child	0
Because the inhaler was not working for your child	1 (1.0%)
Because you did not properly understand the doctor’s instructions on how to apply it	1 (1.0%)
Because you thought the method of application was too complicated	0
Because your child was not cooperating with the administration	6 (6.1%)
Because the inhaler was empty	3 (3.1%)
Because you felt your child was getting worse	3 (3.1%)
Other reasons	11 (11.2%)

**Figure 2:**
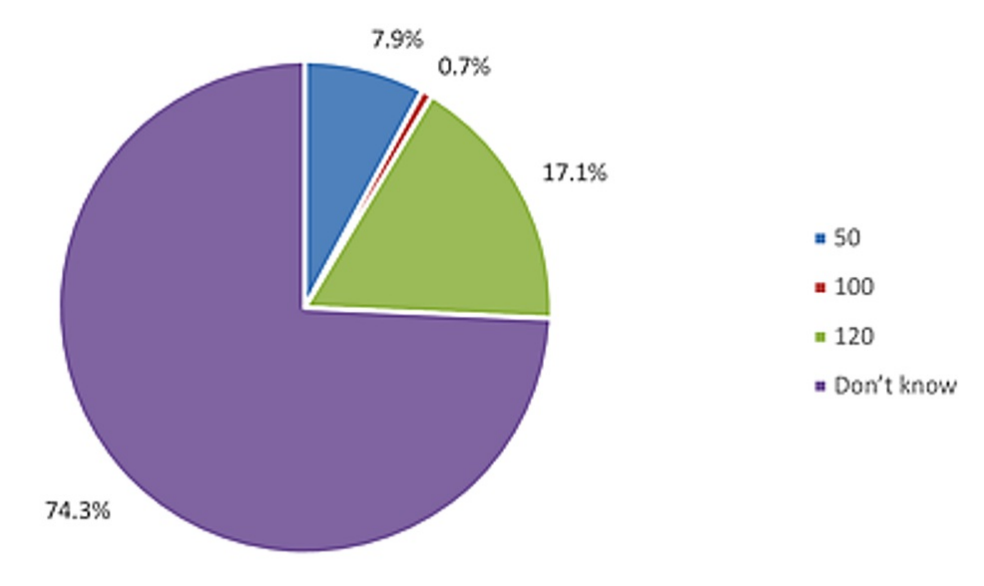
Knowledge about the number of doses present in each fluticasone propionate or fluticasone-salmeterol inhaler

In evaluating the knowledge of participants regarding the number of doses present in each fluticasone propionate or fluticasone-salmeterol inhalers, we found that the majority of participants (74.3%) did not know the number of doses present in the inhaler; only 17.1% indicated knowledge of 120 doses, followed by 7.9% indicating knowledge of 50 doses (Figure 2).

We then looked at the relationship between the level of adherence and the gender as well as the age group; we found that males were more adherent to asthma controller therapy than females; however, the difference was not statically significant (p=0.504). Likewise, the difference in the level of adherence among different age groups was not statically significant (p=0.942) (Table 4). Similar to the age and gender, the differences regarding both the knowledge about the doses of inhaled corticosteroids [fluticasone propionate or fluticasone-salmeterol inhalers (X^2^=0.929)] and the answer to the question “Any doses of the inhaler given to any person other than the child’’ (p=0.818 and p=0.052, respectively) (Table 5) did not reach statistical significance when compared to the adherence to the asthma controller medication.

**Table 4:** Relationship between the level of adherence to asthma controller therapy and the basic demographic data of the patients with asthma (n=152)

Factor	Good, n (%) (n=25)	Poor, n (%) (n=127)	X^2^	P-value
Gender				
Male	17 (68.0%)	74 (58.3%)	0.823	0.504
Female	8 (32.0%)	53 (41.7%)		
Age group				
<2 years	5 (20.0%)	26 (20.5%)	0.393	0.942
2–5 years	9 (36.0%)	41 (32.3%)		
6–10 years	10 (40.0%)	51 (40.2%)		
11–14 years	1 (4.0%)	9 (7.1%)		

**Table 5:** Relationship between the level of adherence to asthma controller therapy and the knowledge about the doses as well as the doses given to any person (n=152)

Factor	Good, n (%) (n=25)	Poor, n (%) (n=127)	X^2^	P-value
Any doses of the inhaler given to any person other than your child				
Yes	0	17 (13.4%)	3.768	0.052
No	25 (100%)	110 (86.6%)		
Knowledge of doses present in each fluticasone propionate or fluticasone-salmeterol inhalers before its use				
50	1 (4.0%)	11 (8.7%)	0.929	0.818
100	0	1 (0.80%)		
120	5 (20.0%)	21 (16.5%)		
Do not know	19 (76.0%)	94 (74.0%)		

## Discussion

To the best of our knowledge, this study is the first to be conducted in the city of Majmaah to assess the adherence to asthma controller therapy among children. There is significant variability among reports assessing the adherence to the asthma medications in the literature [11,19,20]. In a systematic review, it was estimated that 30-70% of prescribed doses of inhaled corticosteroids were taken by children with asthma [11]. In the US, a large randomized trial that spanned over two years assessed the adherence to inhaled corticosteroids in children and the effectiveness of computerized speech recognition telephone calls to improve adherence. It was found that the mean adherence rate was about 36% and was significantly higher in the intervention group [19]. Another study evaluated the adherence to oral asthma medication using an electronic monitoring device and found that the mean adherence was 64% [20]. Interestingly, our study showed the adherence level to be very poor with only 16.4% of the participants reporting adherence to the asthma treatment. Although there is variability in the literature regarding adherence to asthma medications, our finding is lower than that of the previous reports.

A recent study conducted in Brazil evaluated the adherence rate to asthma medications among children and adolescents with moderate asthma by using the inhaler’s dose counter during follow-up visits, and it found good adherence of up to 87.7% among participants, which had helped lead to asthma control [21]. A cross-sectional study conducted in Saudi Arabia studied the adherence among children by using the Morisky Medication Adherence Scale and found an adherence rate of 64.9% to asthma medication, which is similar to what had been reported in the past [22]. However, the adherence was evaluated using prospective objective measures, unlike our study where we assessed the adherence retrospectively, which probably underestimated the rate of adherence due to parents' recall bias.

Prior studies have suggested that nonadherence to asthma medications are possibly caused by multiple factors such as a lack of access to asthma medications, parents forgetting to administer the asthma medications to their children, fear of the possible side effects of the inhaled corticosteroids, and poor parental recognition and lack of awareness about asthma symptoms and asthma medications [23]. Burgess et al. [24] evaluated the barriers of adherence among young children and found that parents forgetting to give inhaled corticosteroid was one of the principal barriers. This finding is consistent with our study, in which ‘’forget to give inhaled corticosteroid to your child’’ was the most common reason for reduced adherence to asthma medications in our participant cohort (55.1%). Two studies conducted in the Middle East found that about half of their participants reported a fear of inhaled corticosteroid’s side effects [25]. A similar finding was observed in a study conducted in Saudi Arabia [26], in which the majority of parents (60.3%) were concerned about the side effects of inhaled corticosteroids and about one-third of the parents were concerned about the potential dependency on asthma inhalers. On the contrary, in our study, only 7% of the parents were afraid that the inhalers would be harmful to their children, and only 1% of the parents were concerned about their children becoming dependent on inhaled corticosteroids. This significant discrepancy between our data and the prior report regarding some of the barriers of adherence to asthma medications is probably related to the difference in sample sizes and possibly the interpretation of the questions by our study participants.

The age group the children belong to may also have an influence on their adherence to asthma medications. In children who were less than five years of age, the adherence is dependent on the parents and their knowledge of asthma as well as their beliefs/perceptions regarding asthma medications. On the other hand, other factors may influence the adherence among school-age children as these children are often away from their parents when they are at school and may get exposed to new triggers at school [27]. Prior studies have suggested that nonadherence is highest among older children and adolescents [28]. A study in the US assessed the adherence to asthma medications and reported that young children appeared to be more adherent compared to older children [29]. In our study, there were no significant differences between different age groups in the rate of adherence to asthma medications (p=0.942). However, a study by Halwani et al. [26] found that children younger than 12 years of age have poor adherence to asthma medication and followed improper inhaler device techniques compared to the older ager group.

Limitations

One of the limitations of our study is the risk of recall bias since the parents of the children with asthma who participated were asked about the adherence to the asthma medications during the prior 15 days. Secondly, the study was conducted in a single center and therefore the results cannot be generalized to the overall Saudi population.

## Conclusions

Our study demonstrated that the overall adherence level to the asthma controller therapy was poor among children. Parents forgetting to give asthma medications was found to be the most common reason for nonadherence in this study. Different age groups, gender, and parental concern about inhalers’ side effects were less likely to influence the level of adherence. This study provides an initial step toward gaining a better awareness about the significant issue of nonadherence to asthma medication and the need to implement steps to decrease the rate of consequent asthma exacerbation. Larger, nationwide studies are needed to more thoroughly assess the adherence to asthma medications and implement interventions that can improve the rates of adherence.
